# Branched-chain amino acids inhibit the TGF-beta-induced down-regulation of taurine biosynthetic enzyme cysteine dioxygenase in HepG2 cells

**DOI:** 10.1007/s00726-014-1693-3

**Published:** 2014-02-20

**Authors:** Asami Hagiwara, Sonoko Ishizaki, Kenji Takehana, Shoji Fujitani, Ichiro Sonaka, Hideo Satsu, Makoto Shimizu

**Affiliations:** 1 Research Institute, Ajinomoto Pharmaceuticals Co. Ltd., 1-1 Suzuki-cho, Kawasaki-ku, Kawasaki, 210-8681 Japan; 2 Department of Applied Biological Chemistry, Graduate School of Agriculture and Life Sciences, The University of Tokyo, 1-1-1 Yayoi, Bunkyo-ku, Tokyo, 113-8657 Japan

**Keywords:** Branched-chain amino acid, Cysteine dioxygenase, Taurine, Transforming growth factor-β, Liver cirrhosis

## Abstract

Taurine deficiency has been suggested to contribute to the pathogenesis and complications of advanced hepatic diseases. The molecular basis for a low level of taurine associated with hepatic failure is largely unknown. Using carbon tetrachloride (CCl_4_)-induced cirrhotic rat model, we found that the activity and expression of cysteine dioxygenase (CDO), a rate-limiting enzyme in taurine synthesis, were significantly decreased in the liver of these rats. To investigate the underlying mechanisms for the suppression, we examined the effects of pathological cytokines on CDO expression in human hepatoma HepG2 cells. Among the several cytokines, transforming growth factor-β (TGF-β), one of the key mediators of fibrogenesis, suppressed *Cdo1* gene transcription through the MEK/ERK pathway. Finally, we further examined potential effects of branched-chain amino acids (BCAA) on CDO expression, as it has been reported that oral BCAA supplementation increased plasma taurine level in the patients with liver cirrhosis. BCAA, especially leucine, promoted *Cdo1* gene transcription, and attenuated TGF-β-mediated suppression of *Cdo1* gene expression. These results indicate that the low plasma level of taurine in advanced hepatic disease is due to decreased hepatic CDO expression, which can be partly attributed to suppressive effect of TGF-β on *Cdo1* gene transcription. Furthermore, our observation that BCAA promotes *Cdo1* expression suggests that BCAA may be therapeutically useful to improve hepatic taurine metabolism and further suppress dysfunctions associated with low level of taurine in hepatic diseases.

**Electronic supplementary material:**

The online version of this article (doi:10.1007/s00726-014-1693-3) contains supplementary material, which is available to authorized users.

## Introduction

Taurine, 2-aminoethanesulfonic acid, is an end product of sulfur containing amino acids (SAA) metabolism with various physiological roles including conjugation with bile acids, stabilization of the cellular plasma membrane, anti-oxidant effects, osmoregulation, detoxification, and neuroprotective effects (Huxtable 1992; Wu et al. 2005). Decreased level of taurine in liver failure is suggested to be contributing to the development of liver failure. For example, taurine transporter knockout mice which exhibit significantly low levels of taurine in plasma and tissues develop chronic hepatitis and liver fibrosis (Warskulat et al. 2006). Taurine supplementation to CCl_4_-induced hepatic fibrotic model rats prevents fibrosis by reducing oxidative stress (Miyazaki et al. 2005). A decreased plasma taurine level in cirrhosis is proposed to be related to occurrence of muscle cramps (Yamamoto 1994, 1996; Yamamoto et al. 1994; Miyazaki et al. 2004), and the taurine supplementation reduced the frequency of muscle cramps (Matsuzaki et al. 1993; Yamamoto 1994, 1996; Yamamoto et al. 1994). Despite that the several evidences suggest a pathophysiological relationship between taurine deficiency and hepatic failure and its associated complications, the underlying mechanisms for this phenomenon remain to be known.

In the present study, using both an animal model and cultured cell experiments, we show that the main reason for taurine deficiency in cirrhosis is decreased expression of its biosynthetic enzyme, CDO. CDO catalyzes the rate-limiting step in the pathway of taurine biosynthesis (Stipanuk 2004). We also show that *Cdo1* gene expression is suppressed by TGF-β, a typical pathogenic cytokine involved in fibrosis. Finally, we show that the decreased *Cdo1* expression can be rescued by BCAA supplementation, which is often used as a treatment for hepatic disease patients.

## Materials and methods

### Animals

Male Sprague–Dawley rats (Charles River Japan, Yokohama), 7-week old, were maintained in an air conditioned room with a 12-h dark/light cycle. They had free access to a standard diet [Charles River Formula-1 (CRF-1); Oriental Yeast, Tokyo] and water. Liver cirrhosis was induced by repeated injections of CCl_4_ according to the standard method of Proctor and Chatamra (Proctor and Chatamra 1983). In brief, CCl_4_ mixed with an equal volume of olive oil was injected subcutaneously twice a week at a dose of 1 ml/kg body weight. To enhance susceptibility to CCl_4_, 0.05 % sodium phenobarbital was given in drinking water from a week prior to the first CCl_4_ injection to the end of the experiment. After 24 weeks of CCl_4_ administration, plasma concentrations of albumin ranged from 2.3 to 3.8 g/dl (Normal rats average 3.9 g/dl). Rats with plasma albumin concentrations below 3.0 g/dl were considered to be cirrhotic rats. Liver cirrhosis was confirmed by Azan-Mallory staining of liver tissue (Fig. S1). The animal facilities and protocol were reviewed and approved by the Institutional Animal Care and Use Committee of Ajinomoto Co., Inc.

### Cell culture

HepG2 cells [kindly provided by Dr. Hosokawa (Jissen women’s university, Japan)] were cultured in Dulbecco’s modified eagle medium (DMEM) containing 10 % FCS. The cells were incubated at 37 °C under a 5 % CO_2_ atmosphere. Cells were seeded in collagen coated 6-well plates at a density of 2 × 10^5^ cells/well and were cultured overnight to reach 70 % confluence. Cells were rinsed with PBS and were precultured with serum-free DMEM or amino acid-free DMEM (Ajinomoto Co., Inc.) for 2 h before subsequent experiments. For cytokine experiments, each cytokine (10 ng/ml) was added to DMEM containing 0.1 % BSA.

### Reagents

Recombinant human IL-1β and human TNF-α were purchased from PeproTech EC Ltd, London. Recombinant human IL-8 and human TGF-β were purchased from R&D Systems, MN, USA. Anti-rat CDO serum was a kind gift of Dr. Hosokawa (Jissen women’s university, Japan). All amino acids were from Ajinomoto Co. Inc, Tokyo. BCAA indicates a mixture of leucine (Leu), isoleucine (Ile), and valine (Val) (Leu:Ile:Val = 2:1:1.2 by weight).

### Gene expression analysis

Total RNA was extracted using Isogen (Nippon gene, Tokyo) according to the manufacturer’s instructions. For northern blot analysis, total RNA was fractionated on 1 % formaldehyde-agarose gel and was transferred onto a nylon membrane (Hybond-N, GE Healthcare, UK). The RNA was cross-linked by UV irradiation before hybridization. The membrane was hybridized with radiolabeled specific DNA probes, and the signals on the membrane were quantified using an image analyzing system, FLA-3000 (Fuji Film Inc. Tokyo). For each sample, hybridization to ribosomal protein L21 (RPL21) or glyceraldehyde 3-phosphate dehydrogenase (GAPDH) was used as an internal control. RPL21 was selected for animal study and in vitro experiments with BCAA treatments because its expression level was stable in all conditions while general genes such as GAPDH and β-actin were greatly affected in cirrhotic rats or by BCAA treatment. For quantitative real-time PCR analysis, first-strand cDNA was synthesized from 1.25 μg of total RNA using superscript III (Invitrogen, CA). Real-Time PCR analysis was carried out on an ABI PRISM™ 7700 Sequence Detector using SYBR Green (Applied Biosystems, CA). Primers used to detect human *Cdo1* are forward, 5′-TGA TAC ATG CCATGC CTT TG-3′, and reverse, 5′-CGA AGT TGC ATT TGG AGT TC-3′. Acquired data were analyzed with Sequence Detector v. 1.7 Alias, and the relative mRNA expression level of the *Cdo1* gene was estimated using *RPL21* as the reference gene.

### Western blot analysis

Aliquots of samples (25 μg of cellular protein) in 20 mM HEPES (pH 7.9), 10 mM KCl, 0.1 mM sodium vanadate, 1 mM EGTA, 1 mM EDTA, 0.2 % NP-40, 10 % glycerol were run on 15 % polyacrylamide gels. Proteins were blotted on PVDF membrane and Western blot analysis was performed using a rabbit antiserum against rat CDO.

### CDO enzymatic activity assay

A CDO activity assay was performed as described by Eppler and Dawson (1999), and the resultant CSA concentration of each sample was measured with an amino acid analyzer (Hitachi L-8800) and was quantified using a standard curve.

### Reporter assay

A DNA fragment that contains the region from nt −2,480 to +2,268 [relative to the transcription initiation site (+1)] of the 5′-flanking sequence of the human *Cdo1* gene was inserted into the firefly luciferase expression vector pGL3-basic (Promega, USA) and was used as a reporter plasmid, pGL3-CDO.

HepG2 cells were transfected by the calcium phosphate method with pGL3-CDO and pRL-SV40, an expression plasmid encoding renilla luciferase as an internal control. 12 h later, the cells were treated with or without cytokines and/or amino acids for 24 h. The luciferase activities were quantified using a Dual-Luciferase reporter assay system (Promega, WI) according to the manufacturer’s protocol.

### Data analysis

Differences were analyzed by Student’s *t* test or one-way analysis of variance (ANOVA) followed by Dunnett’s or Tukey’s multiple comparison test. Values of *p* < 0.05 were considered to indicate statistically significant differences.

## Results

### Changes in the expression of major genes of taurine metabolism and enzymatic activity of taurine synthesis in the liver of cirrhotic rats

After 24 weeks of CCl_4_ administration, cirrhotic rats developed hypoalbuminemia and showed a decreased plasma branched-chain amino acids and tyrosine ratio (BTR) (Table 1).

**Table 1:** Characteristics of the cirrhotic liver model

	Control (*N* = 4)	Cirrhosis (*N* = 4)	*p*
Body weight (g)	596.93 ± 13.48	379.45 ± 20.69	<0.001
AST (IU/l)	62.25 ± 6.75	239.00 ± 26.12	<0.001
ALT (IU/l)	37.25 ± 3.12	115.50 ± 11.64	<0.001
Albumin (g/dl)	3.93 ± 0.09	2.45 ± 0.06	<0.001
Plasma amino acids (μM)			
Taurine	317.8 ± 30.1	205.5 ± 25.2	<0.05
Methionine	72.6 ± 9.1	103.5 ± 8.2	0.06
Leucine	163.6 ± 7.3	128.0 ± 14.2	0.06
Isoleucine	98.9 ± 6.0	84.6 ± 7.8	0.19
Valine	192.1 ± 11.7	151.6 ± 17.8	0.10
Phenylalanine	80.4 ± 2.6	100.1 ± 5.5	0.06
Tyrosine	80.2 ± 2.2	100.1 ± 5.5	<0.01
BTR	5.67 ± 0.23	2.23 ± 0.31	<0.001

Values are the mean ± SEM. Statistical differences were assessed by unpaired Student’s *t* test

*AST* aspartate aminotransferase, *ALT* alanine aminotransferase, *BTR* branched-chain amino acids and tyrosine ratio

**Fig. 1:**
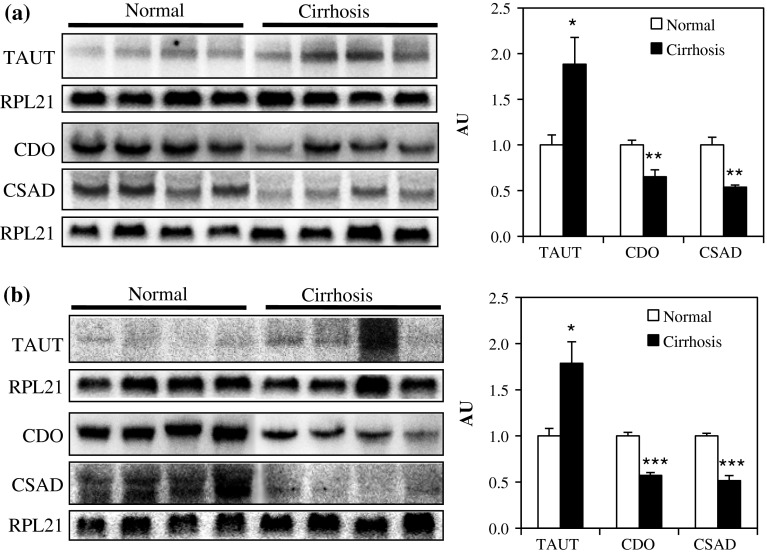
Changes in the expression of major genes associated with Taurine metabolism in cirrhotic rats. Northern blot analysis on TAUT, CDO, and CSAD mRNA expression in the **a** kidney and **b** liver. RPL21 was used as an internal control. *Bar graphs* show the densitometry analysis of band intensities. Results are normalized to the levels of normal rats. Values are the mean ± SEM for four rats, *indicates statistical significance from normal rats (*p < 0.05, **p < 0.01, ***p < 0.001) by Student’s *t* test

**Fig. 2:**
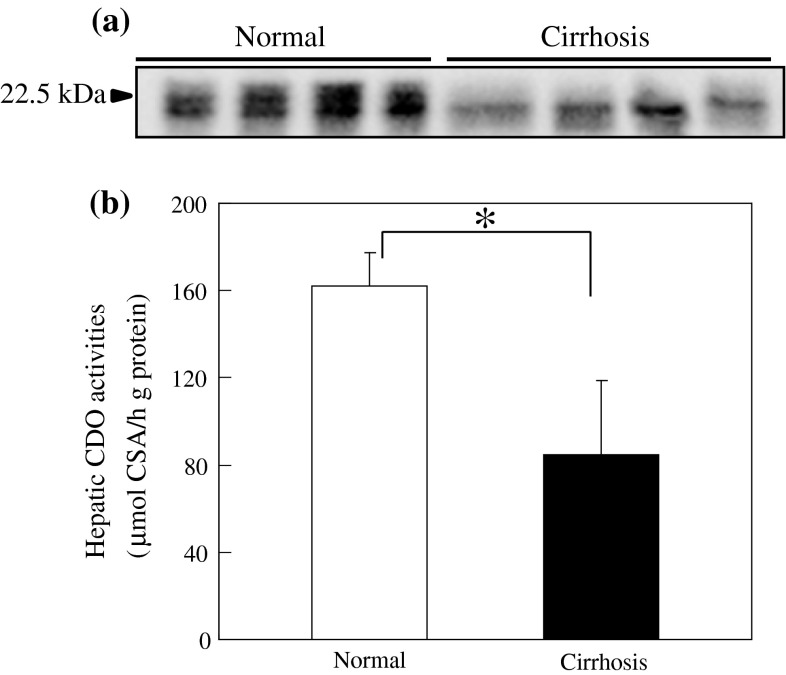
Taurine biosynthetic enzyme CDO is decreased in the liver of cirrhotic rats. **a** Protein expression level and **b** enzymatic activity of CDO in the liver of rats. Each value is the mean ± SEM for four rats. An * indicates significant difference from the control normal rats (*p < 0.05) by Student’s *t* test

We first investigated the mRNA expression of the genes involved in taurine metabolism in the kidney and liver. mRNA levels of taurine biosynthetic enzymes such as CDO and cysteine sulfinic acid decarboxylase (CSAD) were significantly decreased in the kidney (Fig. 1a) and the liver (Fig. 1b) of cirrhotic rats. In contrast, taurine transporter (TAUT) expression in these tissues tended to be increased in cirrhotic rats (Fig. 1). Since the liver is the major tissue that produces taurine, we measured protein amount and the enzymatic activity of hepatic CDO, a rate-limiting enzyme of taurine biosynthesis. Hepatic CDO was significantly decreased both at protein level (Fig. 2a) and activity (Fig. 2b) in cirrhotic rats. These changes were accompanied by a decreased level of plasma taurine in cirrhotic rats (Table 1), suggesting that cirrhotic rats have decreased level of plasma taurine mainly due to a defect in taurine biosynthesis in the liver.

### Effects of inflammatory cytokines on *Cdo1* gene expression in HepG2 cells

To gain further insight into the mechanism of the *Cdo1* gene suppression in cirrhotic rats, we employed in vitro analysis on the regulation of the *Cdo1* gene expression using the human hepatoma cell line, HepG2.

**Fig. 3:**
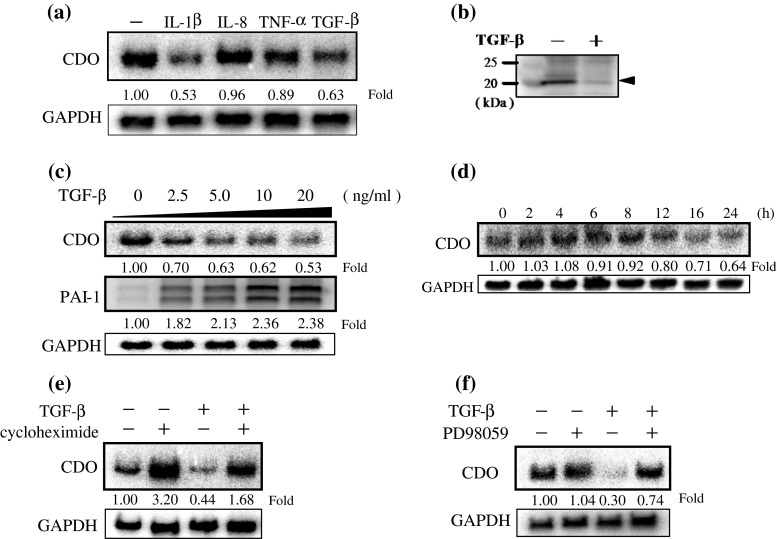
The effects of TGF-β on CDO expression in HepG2 cells. **a** IL-1β, TNF-α and TGF-β suppress CDO mRNA expression in HepG2 cells. The mRNA level of CDO was analyzed by Northern blot analysis. HepG2 cells were treated with serum-free DMEM containing each cytokine (10 ng/ml) for 16 h. GAPDH was used as an internal control. *Each value* represents the relative CDO mRNA level compared with control. **b** Western blot analysis of CDO protein level in HepG2 cells treated with TGF-β (10 ng/ml) for 24 h. **c** TGF-β decreases the CDO mRNA level in a dose-dependent manner. HepG2 cells were treated with increasing amounts of TGF-β at the indicated concentrations for 16 h, and the mRNA levels of CDO expression were quantified by Northern blot analysis. TGF-β responsive PAI-1 mRNA levels were analyzed as a positive control. *Each value* represents the relative mRNA level compared with control. **d** Time course of TGF-β effect on CDO mRNA suppression. CDO mRNA level at each indicated time point was analyzed by Northern blot analysis. *Each value* represents the relative mRNA level compared with time 0. **e** Effects of a protein synthesis inhibitor on TGF-β suppression of CDO mRNA expression. Cells were pre-treated with cycloheximide (20 μg/ml) for 30 min prior to the addition of TGF-β (10 ng/ml). The CDO mRNA levels after TGF-β treatment for 16 h with or without cycloheximide were investigated. *Each value* represents the relative mRNA level to the control in the absence of TGF-β and cycloheximide. **f** Involvement of activation of the MEK/ERK signaling pathway in CDO mRNA suppression by TGF-β. Cells were either pre-treated with PD98059 (20 μM) or not for 30 min prior to the addition of TGF-β. CDO mRNA levels after treatment with TGF-β for 16 h with or without the inhibitor were quantified. *Each value* represents the relative mRNA level to the control in the absence of TGF-β and the inhibitor

The effects of several inflammatory cytokines, which are known to be increased in the course of the development of liver cirrhosis, on CDO mRNA expression in HepG2 cells were analyzed. Cells were treated with several cytokines (IL-1β, IL-8, TNF-α and TGF-β) that had increased expression levels in the livers of cirrhotic rats (Fig. S2). As shown in Fig. 3a, IL-1β and TGF-β significantly decreased CDO mRNA expression. TNF-α moderately suppressed CDO mRNA level as well.

### Characterization of CDO suppression by TGF-β

As TGF-β is a typical inflammatory and fibrogenic cytokine that plays a key role in pathogenesis and development of liver cirrhosis, we further examined the effects of TGF-β on the expression of CDO.

Western blot analysis revealed that TGF-β lowers the CDO protein level as well (Fig. 3b). TGF-β decreased CDO mRNA expression in a dose-dependent manner when it conversely increased mRNA level of plasminogen activator inhibitor-1(PAI-1), a well-known TGF-β target gene (Fig. 3c). Since the suppressive effect of TGF-β on CDO mRNA expression was observed after relatively long time (more than 12 h after stimulation) (Fig. 3d), we speculated that it is regulated through not a direct but an indirect mechanism which requires protein synthesis. Though cycloheximide alone significantly increased the basal CDO mRNA level, it could not suppress the inhibition of CDO mRNA expression by TGF-β (Fig. 3e) (note that the inhibitory effects of TGF-β were about 50 % regardless of the presence or absence of cycloheximide), suggesting that de novo protein synthesis is not required for CDO mRNA suppression by TGF-β. This suppression by TGF-β was inhibited by PD98059, a selective mitogen-activated protein kinase kinase 1 (MEK1) inhibitor (Fig. 3f), but not by p38 and c-jun N-terminal kinase (JNK) inhibitors (data not shown), suggesting that MEK1/extracellular signal-regulated kinase (ERK)-dependent pathway is mediating the suppression.

**Fig. 4:**
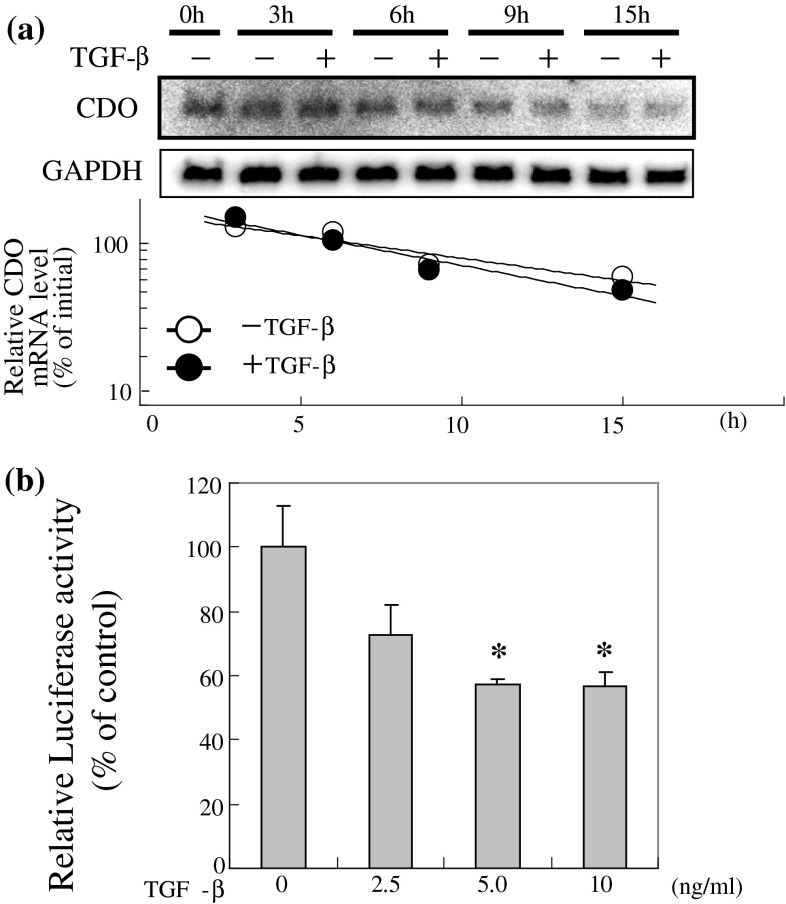
Characterization of *Cdo1* gene suppression by TGF-β. **a** Influence of TGF-β on CDO mRNA stability. Cells were pre-treated with actinomycin D (5 μg/ml) to prevent de novo mRNA synthesis. CDO mRNA level at each indicated time point was analyzed by northern blotting and mRNA degradation rates with or without TGF-β were compared. **b** Effects of TGF-β on transcriptional activity of *Cdo1* gene. HepG2 cells transfected with pGL3-CDO were incubated with increasing amounts of TGF-β for 24 h. Luciferase activities were quantified as described in “Materials and methods”. Each value is the mean ± SD represented as a percentage relative to the control (without TGF-β). * Corresponds to *p* < 0.01 compared to the control by Dunnet’s test

To clarify the mechanism of down-regulation of CDO mRNA expression by TGF-β, we first analyzed CDO mRNA degradative rates. The rates of CDO mRNA degradation following actinomycin D treatment were unchanged with TGF-β treatment (Fig. 4a). The effect of TGF-β on the *Cdo1*gene transcription rate was then analyzed. Using a reporter construct containing the promoter and intron 1 regions of the *Cdo1* gene, we found that TGF-β decreased the *Cdo1* gene transcriptional activity in a dose-dependent manner (Fig. 4b).

### Effects of BCAA on *Cdo1* gene expression

After we found that a key regulatory enzyme of taurine biosynthesis is down-regulated in chronic hepatic disease due in part to a suppressive effect of pathogenic cytokine, TGF-β, on *Cdo1* gene expression, we next examined whether this down-regulation could be reversed by any treatment. Our primary interest was the effect of BCAA treatment, because BCAA supplementation therapy is widely used to improve liver function in patients with chronic hepatic disease in Japan. Indeed, there is a clinical report that chronic BCAA supplementation increased the circulating taurine level in cirrhotic patients (Goto et al. 2001).

**Fig. 5:**
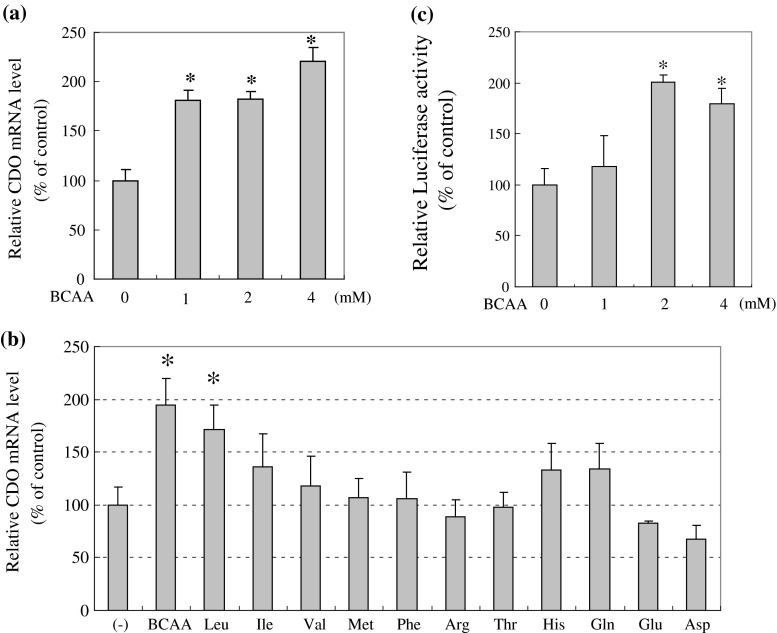
Effects of BCAA and other amino acids on CDO mRNA expression. **a** HepG2 cells were treated with increasing doses of BCAA mixture consisting of Leu, Ile, Val in a ratio of 2:1:1.2 for 16 h. CDO mRNA expression levels were quantified by RT-PCR as described in “Materials and methods”. **b** Cells were treated with each amino acid at a dose of 4 mM for 16 h. CDO mRNA expression levels were analyzed by the same method as indicated in **a**. **c** Transfection and luciferase assay were performed as described in the legend of Fig. 4b. The cells were starved of amino acids for 2 h and then increasing amounts of a mixture of BCAA were added to the culture medium for up to 24 h. Each value is the mean ± SD represented as a percentage relative to the control (without BCAA). * Corresponds to *p* < 0.05 compared to the control

To clarify the effect of BCAA on CDO mRNA expression, we have investigated the level of CDO mRNA expression in HepG2 cells with or without BCAA. As shown in Fig. 5a, BCAA promoted CDO expression in a dose-dependent manner. To explore which amino acid of BCAA is most functional and whether this effect is specific to BCAA, cells were treated with each individual BCAA or several other amino acids. Among the three BCAA, Leu promoted the CDO mRNA level about 1.8-fold above the control. No other amino acids had the same effect as Leu (Fig. 5b).

Then, we evaluated the effect of BCAA on promoter activity of the *Cdo1* gene. HepG2 cells were transfected with reporter gene and cultured with increasing amounts of BCAA for 24 h. BCAA induced the promoter activity of the *Cdo1* gene in a dose-dependent fashion (Fig. 5c), with the maximal level almost double the control value.

### Comparison of the effects of BCAA and TGF-β on the transcription of the *Cdo1* gene

**Fig. 6:**
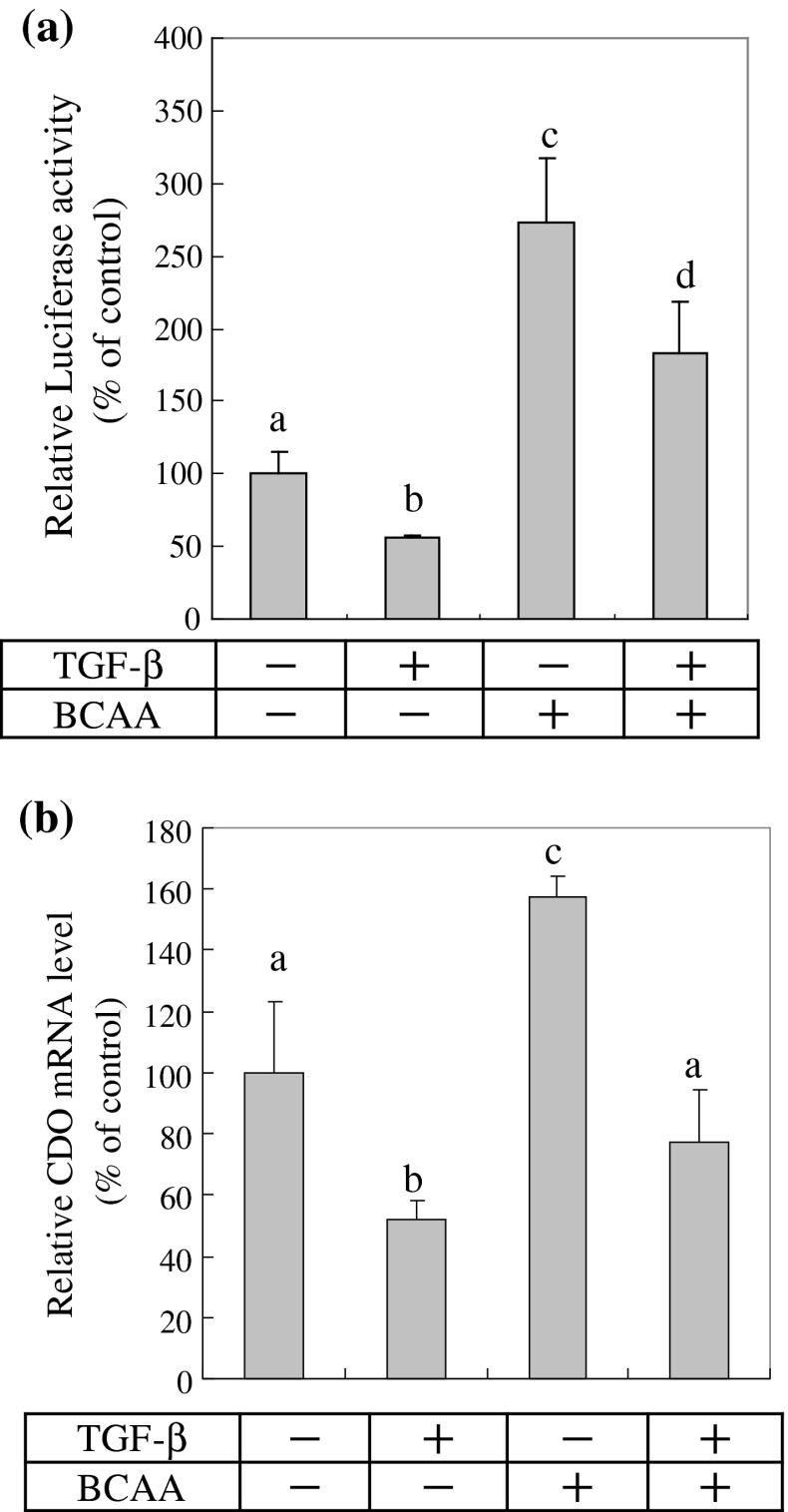
Bidirectional effects of TGF-β and BCAA on *Cdo1* gene expression. **a** Transfection and luciferase assay were performed as described in the legend of Fig. 4b. Cells were incubated in the presence or absence of TGF-β (10 ng/ml) and/or BCAA (4 mM). The luciferase activities were quantified 24 h later. **b** Cells were treated with TGF-β (10 ng/ml) and/or BCAA (4 mM) for 16 h. CDO mRNA expression levels were analyzed by the same method as indicated in Fig. 5a. Each value is the mean ± SD represented as a percentage relative to the control (without TGF-β and BCAA). *Letters above the bars* indicate significant difference at *p* < 0.05, one-way ANOVA followed by Tukey’s test

Finally, we explored the regulation of *Cdo1* promoter activity comparing the effects of BCAA and TGF-β. As shown in Fig. 6a, the suppressive effect of TGF-β on *Cdo1* transcription is inhibited in the presence of BCAA. When BCAA was added to the culture medium together with TGF-β, the promoter activity of *Cdo1* was increased twofold over the control value. The same result was obtained by RT-PCR analysis of the CDO mRNA expression (Fig. 6b). These results indicate that the inducible effect of BCAA on *Cdo1* gene transcription prevails over the suppressive effect of TGF-β.

## Discussion

It is well known that the metabolism of SAA is impaired in patients with advanced liver diseases (Iber et al. 1957; Ferenci and Wewalka 1978; Byrd et al. 1993; Avila et al. 2000; Tietge et al. 2002). The levels of plasma methionine (Met) and homocysteine (Hcy) are elevated while those of glutathione and taurine are declined (Marchesini et al. 1992; Bianchi et al. 2000). In a previous study exploring the relationship between plasma amino acid profiles and liver fibrosis, it is clearly demonstrated that abnormal SAA patterns in patients with chronic hepatitis C are correlated with the progression of liver fibrosis (Zhang et al. 2006). The plasma Met level increased along with the development of the fibrotic stage, and the plasma taurine level decreased in the advanced stages of fibrosis.

The molecular basis for altered SAA metabolism in patients with chronic liver failure is mainly ascribed to abnormal gene expressions of the enzymes involved in SAA metabolism. For example, the amounts of almost all of the main enzymes involved in SAA metabolism such as methionine adenosyltransferase (MAT), glycine-N-methyltransferase, betaine homocysteine methyltransferase (BHMT), cystathionine beta synthase (CBS), and methionine synthase (MS) are significantly reduced, as determined from the mRNA expression level, in cirrhotic patients (Avila et al. 2000; Garcia-Tevijano et al. 2001). Another study demonstrated that clustered abnormalities were found in expression of the genes associated with SAA metabolism in the liver of dimethylnitrosamine (DMN)-induced fibrosis model (Takahara et al. 2006). Among them, down-regulated expressions of CDO and CSAD were strongly associated with the development of the fibrotic stage. In the present study, we also found that the enzymatic activity and the expression of CDO are severely decreased in cirrhotic rat model livers. This is thought to be the main reason for the decreased plasma taurine levels in animals with cirrhosis.

CDO is a well-known key regulator for the synthesis of taurine, and the regulation of its activity has been studied in detail. The main regulatory mechanism for CDO enzymatic activity operates at the posttranslational level. In normal physiological states, postprandial high levels of Met or Cys enhance the activity of CDO by inhibiting its degradation through the ubiquitin proteasome pathway (Stipanuk et al. 2004; Dominy et al. 2006). In the case of cirrhosis, however, CDO activity is significantly decreased in spite of an elevated Met level. It seems that the regulation of expression at mRNA level, rather than protein level, is crucial for CDO expression in cirrhotic state. As the CDO gene expression is suppressed along with the development of fibrosis, we were interested to know whether pathogenic inflammation involved in an early stage of fibrogenesis is associated with CDO gene suppression. Therefore, we examined the effects of several pathological cytokines and found that IL-1β, TNF-α and TGF-β down-regulate CDO mRNA level. The observed effect of IL-1β was consistent with a finding by Dr. Hosokawa (personal communication). The effects of TNF-α and TGF-β were consistent with a previous report (Wilkinson and Waring 2002) which showed that these cytokines decreased CDO protein level in neuronal and hepatic human cells by unknown mechanisms. In the present study, we focused on the effect of TGF-β, a key mediator of fibrogenesis in the development of cirrhosis (Gressner et al. 2002), and demonstrated that *Cdo1* gene transcription is suppressed by TGF-β. As mentioned in introduction, taurine deficiency contributes to the development of chronic hepatic failure. Thus, the observation suggests that the suppression of *Cdo1* gene by TGF-β and subsequent decline of taurine level may be important for the development of cirrhosis.

TGF-β signaling pathway has been implicated in diverse cellular processes by regulating a wide range of genes. TGF-β signals through Smad-dependent and Smad-independent pathways (Derynck and Zhang 2003). We speculate that TGF-β-mediated suppression of *Cdo1* gene transcription is Smad-independent, as overexpression of Smad2 and Smad3 had no effect in our preliminary experiments. Mitogen-activated protein kinases (MAPKs) are implicated in Smad-independent TGF-β signaling. Among them, we showed that ERK is involved in *Cdo1* gene suppression. However, the downstream molecular mechanisms remain unclear. Recently, it has been reported that many of the TGF-β target genes are regulated by DNA methylation in some cancer cells (Matsumura et al. 2011; Zhang et al. 2011). It is of interest to note that the TGF-β signaling pathway mediates DNA methylation by inducing DNA methyltransferase in ERK-dependent mechanism (Zhang et al. 2011). Moreover, *Cdo1* has been reported as a tumor suppressor gene which is suppressed by DNA methylation (Brait et al. 2012). Taken together, it may be interesting to study whether *Cdo1* gene suppression by TGF-β is mediated by DNA methylation.

The effects of BCAA on taurine metabolism were of interest to us from some clinical evidence. Cirrhotic patients are often prescribed supplementation with these amino acids in Japan. Some clinical reports have demonstrated that oral supplementation of BCAA to patients successfully suppressed the occurrence of muscle cramps (Goto et al. 2001; Sako et al. 2003). In one case, BCAA supplementation resulted in an increased circulating taurine level together with decrease in methionine level (Goto et al. 2001). From these observations, we assume that the effect of BCAA on ameliorating muscle cramps can be attributed in part to improved taurine biosynthesis in the liver. Indeed, we showed that BCAA, especially Leu, promoted *Cdo1* gene expression through up-regulation of its transcriptional activity. We also showed that TGF-β-mediated *Cdo1* suppression could be recovered by BCAA administration. Although the precise mechanisms for BCAA-mediated up-regulation of *Cdo1* gene expression remain to be known, these results suggest the potential of BCAA to ameliorate the impaired taurine metabolism in the cirrhotic state.

To summarize, in an effort to clarify the mechanism for taurine deficiency in the chronic hepatic disease, we have demonstrated that the taurine synthetic rate is significantly decreased because of a reduced *Cdo1* gene expression. A common pathogenic cytokine, TGF-β, was found to suppress transcription of the gene, while BCAA antagonizes this suppression, suggesting a potential pharmacological significance of BCAA supplementation to the patients with hepatic failure that have defects in taurine metabolism.

## Electronic supplementary material

Below is the link to the electronic supplementary material.

Supplementary material 1 (PPTX 1434 kb)
