# Supplementary material for: Branched-chain amino acids inhibit the TGF-beta-induced down-regulation of taurine biosynthetic enzyme cysteine dioxygenase in HepG2 cells
Source: Amino Acids. 2014 Feb 20;46(5):1275–83. doi: 10.1007/s00726-014-1693-3 (PMC3984414; doi:10.1007/s00726-014-1693-3)
Supplement: Supplementary file 1 — Supplementary material 1 (PPTX 1434 kb) [file 726_2014_1693_MOESM1_ESM.pptx]

## Slide 1
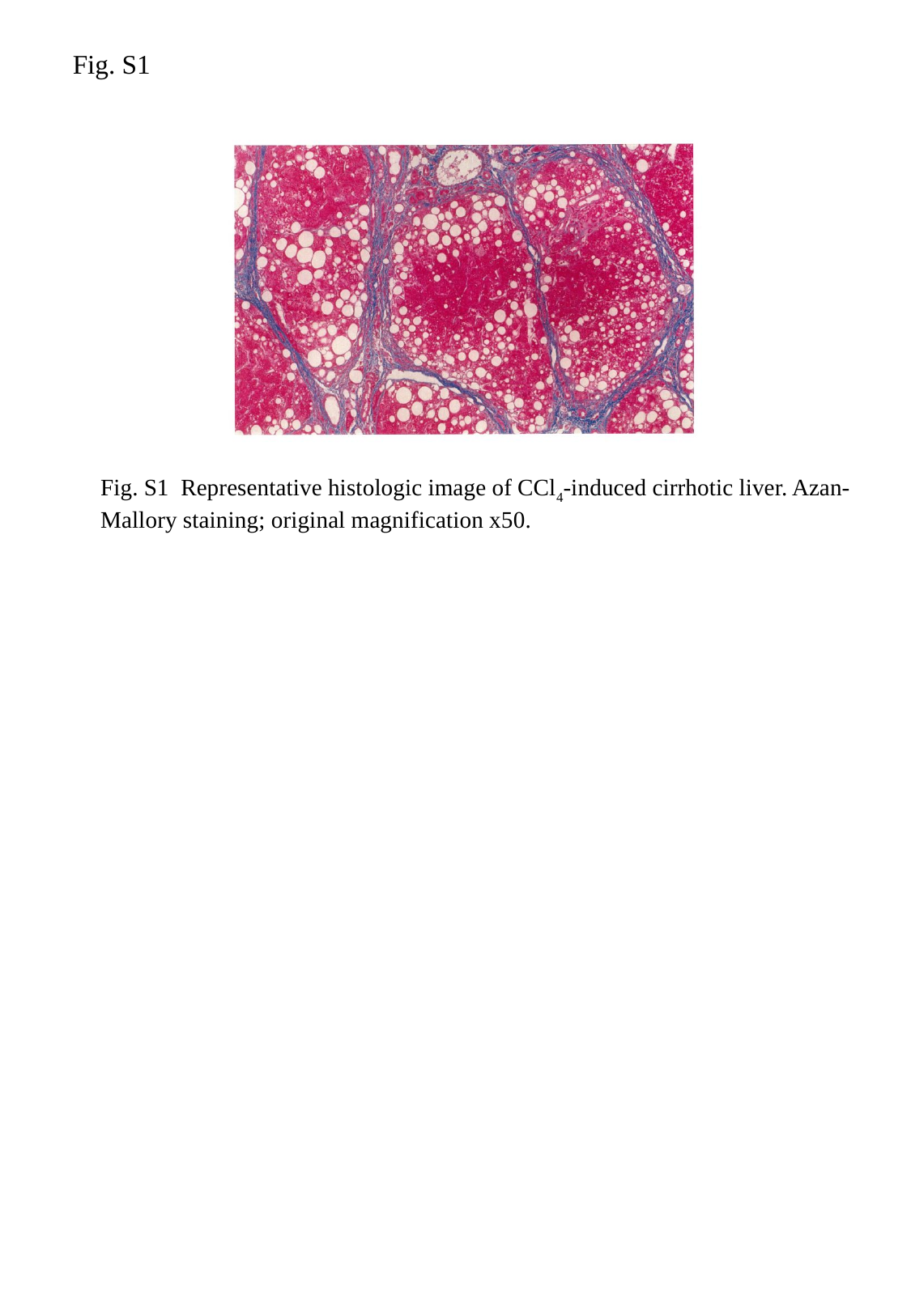

Fig. S1
Fig. S1 Representative histologic image of CCl4-induced cirrhotic liver. Azan-Mallory staining; original magnification x50.

## Slide 2
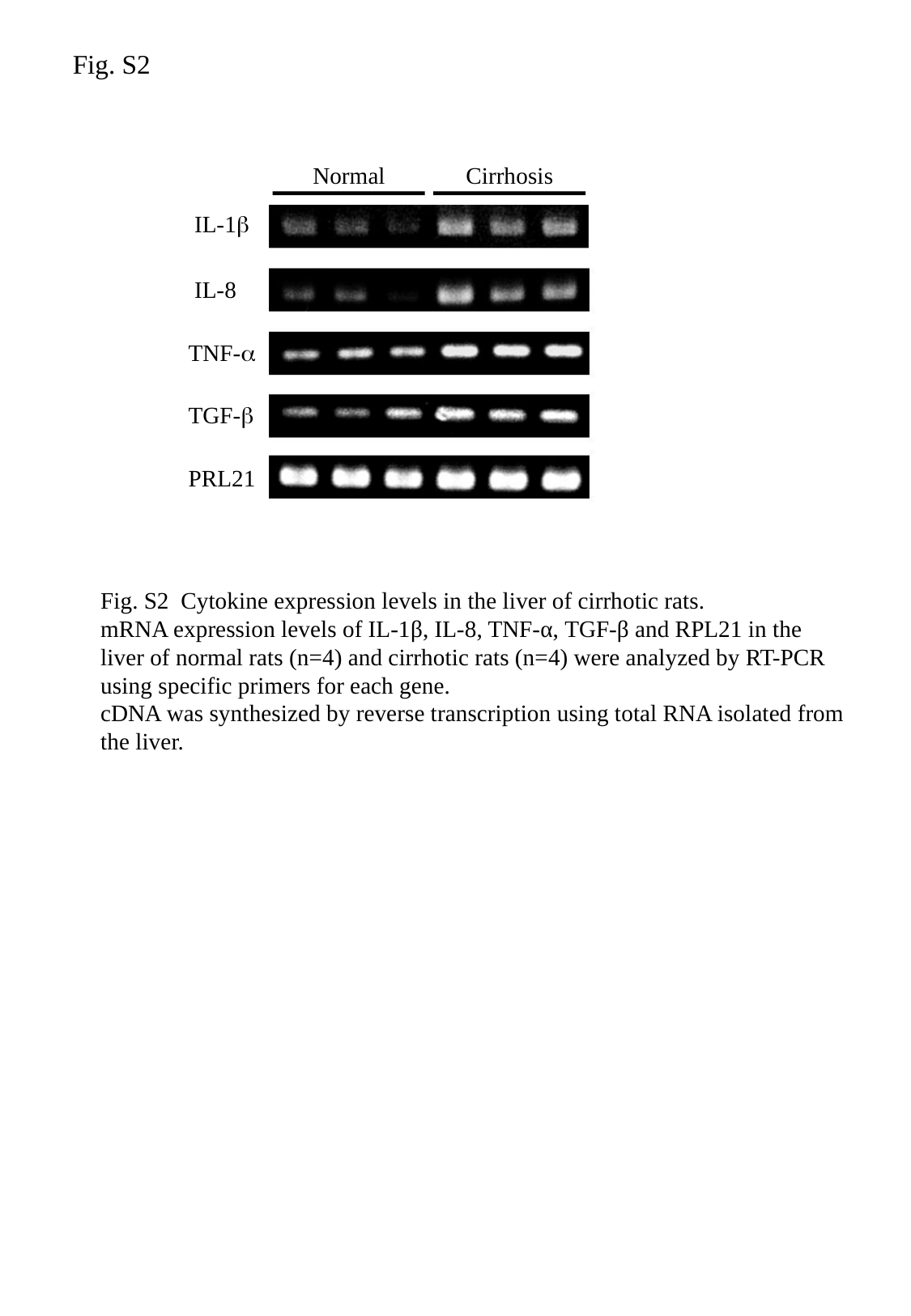

Fig. S2
Cirrhosis
Normal
 IL-1b
 IL-8
TNF-a
TGF-b
PRL21
Fig. S2 Cytokine expression levels in the liver of cirrhotic rats.
mRNA expression levels of IL-1β, IL-8, TNF-α, TGF-β and RPL21 in the liver of normal rats (n=4) and cirrhotic rats (n=4) were analyzed by RT-PCR using specific primers for each gene.
cDNA was synthesized by reverse transcription using total RNA isolated from the liver.
